# Evolutionary characteristics of SARS-CoV-2 Omicron subvariants adapted to the host

**DOI:** 10.1038/s41392-023-01449-w

**Published:** 2023-05-23

**Authors:** Haijun Tang, Yun Shao, Yi Huang, Shigang Qiao, Jianzhong An, Ruhong Yan, Xin Zhao, Fang Meng, Xiaohong Du, F. Xiao-Feng Qin

**Affiliations:** 1 Institute of Systems Medicine, Chinese Academy of Medical Sciences & Peking Union Medical College; Suzhou Institute of Systems Medicine, Suzhou, 215123 China; 2 Shengli Clinical Medical College, Fujian Medical University; Center for Experimental Research in Clinical Medicine, Fujian Provincial Hospital, Fuzhou, 350001 China; 3 Institute of Clinical Medicine Research, Suzhou Hospital, Affiliated Hospital of Medical School, Nanjing University, Suzhou, 215153 China

**Keywords:** Microbiology, Molecular biology, Immunology

**Dear Editor**,

As the COVID-19 epidemic proceeds, new variants of SARS-CoV-2 continue to emerge.^1^ Particularly the B.1.1.529 (Omicron) variant emerged and spread globally, surpassing the previously dominant B.1.617.2 (Delta) variant. Moreover, the original Omicron variant continued to mutate into numerous Omicron sublineages. Nonetheless, the infectious and immune characteristics of newly-emerged Omicron sublineages that have evolved to adapt to the host remain unclear. Here, we systematically investigated the infectivity, proteolytic activation, viral entry pathway, membrane fusion, and sensitivity to antibody neutralizing of the predominant Omicron sublineages.

The Omicron sublineages exhibit mutations in their spike (S) proteins, especially in the receptor binding domain (RBD) (Supplementary Fig. S1a). These variations may lead to changes in the conformation of S protein, affecting its interaction with the receptors or neutralizing antibodies. To investigate the potential infectivity of Omicron sublineages in susceptible cell types, we employed a vesicular stomatitis virus (VSV)-based pseudovirus system. Our findings indicated that pseudoviruses carrying BA.1.1 and BA.3S proteins exhibited similar infectivity as BA.1 (Fig. 1a). However, pseudoviruses carrying the S protein of the BA.2 and its descendants (BA.2.11, BA.2.12.1, BA.2.13, BA.2 + L452Q, and BA.4/5) exhibited increased infection compared to BA.1. Furthermore, BA.2.12.1, BA.2 + L452Q, BA.4/5, BF.7, BQ.1, BQ.1.1, XBB and XBB.1.5 sublineages exhibited stronger infectivity than BA.2 (Fig. 1a, b, Supplementary Fig. S1b). These findings suggested that newly-emerged Omicron sublineages have a trend of gaining higher cellular infectivity.

**Fig. 1:**
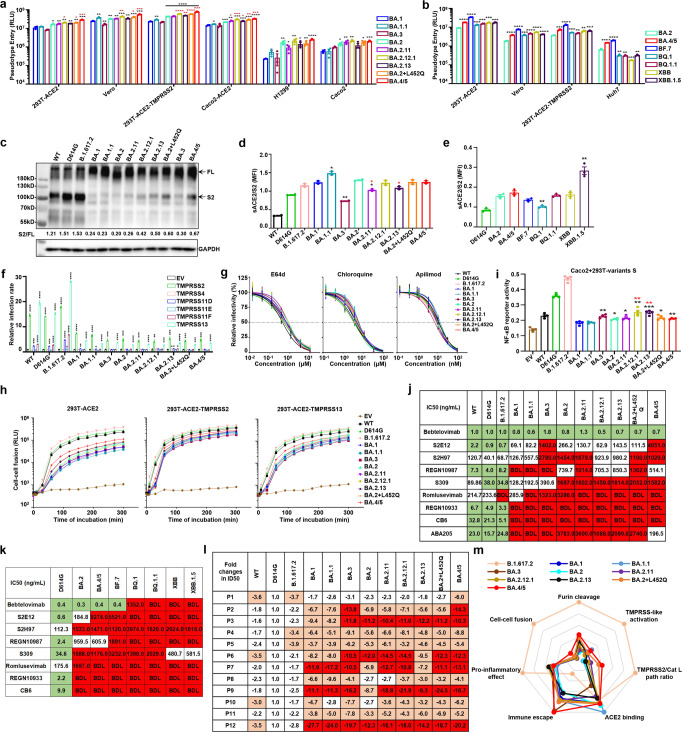
Evolutionary characteristics of SARS-CoV-2 Omicron variants adapted to the host. **a**, **b** Infectivity of Omicron sublineages pseudoviruses in different cells. **c** Hydrolytic cleavage of variants S proteins in 293T cells. Shown are representative blots from three experiments. **d** The binding activity of variants S proteins to soluble ACE2, quantified by mean fluorescence intensity (MFI), and normalized by cell surface expressed S protein (*n* = 2). **e** The binding activity of BA.4/5, B.7, BQ.1, BQ.1.1, XBB and XBB.1.5S proteins to soluble ACE2. **f** Infectivity of variants pseudoviruses in 293T-ACE2 cells transiently expressing different transmembrane serine proteases. **g** Endocytosis and protease inhibitors effectively blocked the entry of variants pseudoviruses into 293T-ACE2 cells. 293T-ACE2 cells were pretreated with different concentrations of inhibitors (E64d, Chloroquine and Apilimod) and then infected with variants pseudoviruses. **h** SARS-CoV-2 variants S proteins mediated cell-cell fusion assay. **i** NF-κB reporter activation in Caco2 cells mediated by incubation with 293T cells expressing variants S proteins. **j**, **k** Neutralizing activity of therapeutic neutralizing mAbs against variants pseudoviruses. Green, IC50 < 50 ng/mL; Red, IC50 > 1000 ng/mL; BDL indicates neutralizing activity below detection limit. **l** Fold changes of vaccine sera neutralization activity between variants and D614G pseudoviruses, and “−” represents the decrease of sensitivity of vaccine sera, and “+” represents the increase of sensitivity of vaccine sera. The values are marked in red, indicating that sensitivity is decreased at least 3-fold. **m** Radar chart of Omicron sublineages characteristics. Experiments were done in 3-4 replicates and repeated at least twice. One representative is shown with error bars indicating SEM. In Fig. 1a, d, i, black stars represent statistical differences of all Omicron sublineages compared to the BA.1 strain, while red stars represent statistical differences of all BA.2 sublineages compared to the BA.2 strain. EV indicates empty vector

To explore the mechanism of the elevated infectivity of Omicron sublineages, we first analyzed the proteolytic process of S proteins. The expression level of variants S proteins was similar, while the cleaved bands (S2) showed divergent patterns (Fig. 1c, Supplementary Fig. S2a, b). The cleavage of all Omicron sublineages S protein was significantly weaker than that of the D614G and B.1.617.2 strains. But, within the Omicron lineage, S protein cleavage was slightly enhanced in BA.2.12.1, BA.2.13, BA.2 + L452Q, BA.4/5, BF.7, BQ.1 and BQ.1.1 compared to the BA.2. Next, we analyzed the binding affinity of variants S proteins to soluble ACE2 (sACE2) (Fig. 1d, e, Supplementary Fig. S3a, b). Similar to previous studies,^1^ the Omicron sublineage S protein had a stronger binding affinity to ACE2 than WT and D614G. Compared with BA.2, the binding affinity of BA.2.11, BA.2.13 and BQ.1S protein to sACE2 was slightly weakened, but the binding affinity of XBB.1.5 was significantly enhanced, posing an important factor for the increased infectivity of XBB.1.5 in the population.

To identify the potential regulator of viral entry into host cells for Omicron sublineages, we examined the infectivity of the variants in 293T-ACE2 cells expressing different transmembrane serine proteases. TMPRSS2/11D/11F/13 significantly promoted the infection of WT, D614G and B.1.617.2 strains, but had relatively weak effects on all Omicron sublineages (Fig. 1f). Moreover, BA.3 and all BA.2 descendants were less dependent on TMPRSS2 compared with BA.1 (Supplementary Fig. S4a). To further investigate the underlining mechanisms, we analyzed the selection of viral entry pathways by pretreating 293T/Caco2-ACE2-TMPRSS2 cells with Camostat (TMPRSS-like protease inhibitor) and/or E64d (endosomal cathepsin B and L (CatB/L) inhibitor). Consistent with previous reports,^2,3^ WT, D614G and B.1.617.2 pseudoviruses entered cells mainly through the Camostat-sensitive pathway, in contrast Omicron sublineages entered cells via the E64d-sensitive pathway (Supplementary Fig. S4b, c). On the other hand, the BA.2 + L452Q and BA.4/5 pseudoviruses showed higher sensitivity to Camostat than BA.1. To extend this study, we also evaluated the effect of endocytosis inhibitors Chloroquine, Apilimod and cathepsin inhibitor E64d on the entry of Omicron sublineages into 293T-ACE2 cells. We found that all three inhibitors reduced the entry of variant pseudoviruses in a dose-dependent manner, and the blocking effects were similar for all Omicron sublineages (Fig. 1g).

Enhanced fusion activity mediated by SARS-CoV-2 S protein is an important factor for increased viral infectivity.^4^ We next examined the dynamics of cell-cell fusion mediated by SARS-CoV-2 S and ACE2. WT, D614G and B.1.617.2S proteins showed higher fusion activity and syncytium formation, while all Omicron sublineages S protein exhibited lower membrane fusion activity (Fig. 1h, Supplementary Fig. S5a-d). Notably, the BA.4/5S protein showed slightly enhanced fusion activity compared to the original BA.1. It has been reported that SARS-CoV-2 S/ACE2-mediated cell-cell fusion induce pyroptosis to exacerbate inflammatory responses.^5^ We thus examined Omicron S protein-mediated pro-inflammatory responses, and found that all the Omicron sublineages only slightly promoted NF-κB pathway activation (Fig. 1i). However, compared with BA.1, the activation of NF-κB pathway induced by the BA.3 and BA.2 descendants S proteins was slightly enhanced. Taken together, our findings suggest that certain Omicron sublineages exhibit stronger fusion and pro-inflammatory responses than the BA.1, which may contribute to their distinct pattern of infectivity and pathogenicity.

The large number of mutations found in Omicron S protein leads to the concerns of the effectiveness of existing monoclonal antibodies (mAbs) and vaccines.^5,6^ We thus examined the neutralization susceptibility of Omicron sublineages to various mAbs, and found that most mAbs either failed to neutralize the Omicron sublineage or had significantly reduced neutralizing efficacy (Fig. 1j, k, Supplementary Fig. S6a, b). Similarly, the neutralizing activity of the inactivated vaccine sera against Omicron sublineages was significantly reduced compared to the D614G strain (Fig. 1l, Supplementary Fig. S7a, b). Together, our results suggest that Omicron continues to evolve into a broad range of sublineages with greater and diversified immune evasion capability, which might explain why some fully vaccinated people are still infected with newly-emerged variants.

Cellular infectivity and immune evasion are the key risk factors of newly-emerging SARS-CoV-2 variants. Previous studies have shown that Omicron spikes have lower cleavage rates in cells compared to B.1.617.2 and WT spikes,^7,8^ which is consistent with our study. We also found that the Omicron sublineages continued to evolve into more infectious strains, and its increased infectivity may be related to enhanced cleavage of the S protein. Furin cleavage site locates at the S1/S2 junction of SARS-CoV-2 S protein, which affects the cleavage efficiency of S protein.^9^ Omicron has three mutations near the Furin cleavage site (P681H, H655Y, and N679K), which attenuates S1/S2 cleavage of spike protein.^7,8,10^ The suboptimal cleavage of S1/S2 in Omicron spike reduces TMPRSS2 utilization, leading to inefficient S2’ processing and fusion peptide exposure.^7,10^ Diminished Omicron spike protein-mediated membrane fusion may be associated with attenuated viral pathogenicity. New Omicron sublineages with stronger immune evasion are constantly evolving due to existing immune pressure (Fig. 1m). The L452 mutation in the Omicron BA.2 sublineages has been reported to facilitate the evasion of some antibodies directed against the RBD region, thereby enhancing the immune escape of the virus.^1,6^ We found that the neutralizing activity of most mAbs against the Omicron sublineages decreased. Particularly some newly-emerging strains, such as BA.4/5, BF.7, BQ.1, BQ.1.1, XBB and XBB.1.5 exhibited much greater immune escape capacity. Consistently, Omicron pseudoviruses showed a significantly reduced sensitivity to inactivated viral vaccine sera. These findings highlight the need to intensify continuous monitoring of Omicron variants and assess the effectiveness of therapeutic mAbs and vaccines against newly-emerging SARS-CoV-2 variants. Meanwhile, applying a combination of multiple therapeutic modalities and developing pan-β-coronavirus neutralizing mAbs and vaccines might prove to be practical strategies for the prevention and treatment of currently circulating and future emerging SARS-CoV-2 variants.

## Supplementary information

Supplementary Information-clean version

## Data Availability

The data that support the findings of this study are available from the corresponding author upon reasonable request.
