# Supplementary material for: Evolutionary characteristics of SARS-CoV-2 Omicron subvariants adapted to the host
Source: Signal Transduct Target Ther. 2023 May 23;8:211. doi: 10.1038/s41392-023-01449-w (PMC10204007; doi:10.1038/s41392-023-01449-w)
Supplement: Supplementary file 1 — Supplementary Information-clean version [file 41392_2023_1449_MOESM1_ESM.docx]

**Supplementary Information for**

**Evolutionary characteristics of SARS-CoV-2 Omicron subvariants adapted to the host**

Haijun Tang^1,2^, Yun Shao^1^, Yi Huang^2^, Shigang Qiao^3^, Jianzhong An^3^, Ruhong Yan^3^, Xin Zhao^1^, Fang Meng^1*^, Xiaohong Du^1,3*^, F. Xiao-Feng Qin^1*^

^1^Institute of Systems Medicine, Chinese Academy of Medical Sciences & Peking Union Medical College; Suzhou Institute of Systems Medicine, Suzhou 215123, China

^2^Shengli Clinical Medical College, Fujian Medical University; Center for Experimental Research in Clinical Medicine, Fujian Provincial Hospital, Fuzhou 350001, China

^3^Institute of Clinical Medicine Research, Suzhou Hospital, Affiliated Hospital of Medical School, Nanjing University, Suzhou 215153, China

*Correspondence: Xiaohong Du ([dxl4857@126.com](mailto:dxl4857@126.com)); Fang Meng ([onionmf@163.com](mailto:onionmf@163.com)); F. Xiao-Feng Qin ([fqin1@foxmail.com](mailto:fqin1@foxmail.com))

**This PDF file includes:**

Materials and Methods

Figure S1 to S7

**Materials and Methods**

**Serum samples**

Vaccine sera were obtained from participants who received three doses of inactivated SARS-CoV-2 vaccines (CoronaVac and BBIBP-CorV). Serum samples were collected from participants on day 10 after booster injection and all samples were heat-inactivated at 56°C for 1 h. Relevant experiments regarding SARS-CoV-2 were approved by the Ethics Committee of Suzhou Hospital, Affiliated Hospital of Medical School, Nanjing University (Ethics committee archiving No. IRB2021006). All participants provided written informed consent for the use of their clinical samples.

**Monoclonal antibodies**

All monoclonal antibodies (mAbs) used in this study were screened and produced by AtaGenix. Among them, the antibodies Bebtelovimab, S2E12, S2H97, Romlusevimab, CB6, REGN10933, REGN10987, and S309 were synthesized according to the sequences published in the Protein Data Bank (PDB). Antibodies were re-aliquoted and stored at -70°C to avoid inconsistent results due to repeated freeze-thaw cycles.

**Cell lines**

293T (human, kidney), Vero (African green monkey, kidney), H1299 (human, lung), Caco-2 (human, colon), Huh7 (human, liver), and A549 (human, lung) cells were obtained from American Type Culture Collection (ATCC). 293T-ACE2, Caco-2-ACE2, A549-ACE2, and 293T-ACE2-TMPRSS2 cells were produced by lentiviral mediated gene transduction. All above cell lines were cultured in Dulbecco’s Modified Eagle Medium (DMEM, Gibco) containing 10% fetal bovine serum (FBS, Gibco) and 100 U/mL of Penicillin-Streptomycin solution (Gibco).

**Production of SARS-CoV-2 variants pseudoviruses**

Plasmids with SARS-CoV-2 variants spike (S) were synthesized by Genscript Biotechnology Company. To facilitate packaging of pseudoviruses, we eliminated the last 19 amino acids containing endoplasmic reticulum retention signals in the cytoplasmic tail of S protein. The mutation sites of the variants are shown in supplementary Fig. S1a. Pseudoviruses bearing SARS-CoV-2 S protein were generated as previously described ^1^. Briefly, 293T cells were transfected with pcDNA3.1 vectors encoding SARS-CoV-2 S genes by lipofectamine 3000 (Invitrogen). At 12 h post-transfection, cells were inoculated with G*△G-VSV dual reporter virus (kindly provided by UltraImmune Inc.). After 12 h of incubation, the inoculum was removed and cells were washed three times with PBS. Subsequently, 10 mL of fresh DMEM medium was added to the cell culture dish. After 24 h, the pseudovirus-containing supernatants were collected, centrifuged to remove cellular debris, aliquoted and and stored at -80°C until use.

**Pseudovirus infection assay**

We normalized pseudoviral particles to the same amount using quantitative RT-PCR. For experiments evaluating S protein-driven cell entry, we seeded 3 × 10^4^ target cells into each well of 96-well plates. Then, 100 μL of media containing pseudoviruses was inoculated into the cells. At 24 h post inoculation, cells were lysed with passive lysis buffer (Promega) for 10 min. Afterwards, cell lysates were mixed with luciferase substrate (Promega) and measured using SpectraMax L Microplate Reader (Molecular Devices).

**Detection of SARS-CoV-2 variants S proteins**

To detect the cleavage of the S protein in cells, 293T cells were transfected with expression plasmids encoding SARS-CoV-2 variants S protein. 40 h post transfection, cells were harvested and lysed with RIPA lysis buffer (Beyotime). Next, samples were heated at 96°C for 10 min and subjected to SDS-PAGE and immunoblotting. After protein transfer, PVDF membranes were blocked with 5% skimmed milk solution for 1 h and then incubated with primary antibody overnight. The next day, membranes were incubated with secondary antibody for 1 h and visualized by ChemiDoc MP system (Bio-Rad). The following antibodies were used: GAPDH monoclonal antibody (Proteintech, 1:2000), mouse anti-SARS-CoV-2 spike (Genetex, 1:2000), horseradish peroxidase-linked anti-mouse IgG antibody (Cell Signaling Technology, 1:5000).

**Flow cytometry analysis of soluble ACE2 binding to variants S proteins**

The binding activity of variants S proteins to soluble ACE2 was detected by flow cytometry according to previously reported ^2^. 293T cells were transfected with expression plasmids encoding SARS-CoV-2 variants S proteins with Lipofectamine 3000. After 36 h, cells were separated with trypsin or EDTA, then washed twice with 1 mL staining buffer. Cells were incubated with mouse anti-SARS-CoV-2 spike antibody (Genetex, 1 μg/mL) or recombinant ACE2 protein (Sino Biological, 1 μg/mL) for 1 h. Then, cells were incubated with Brilliant Violet 421-labeled anti-human IgG Fc (Biolegend), PE-labeled anti-human IgG Fc (Biolegend), or PE-labeled anti-mouse IgG (Biolegend) secondary antibodies for 1 h. After washing, cells were resuspended and analyzed with BD LSRFortassa (BD Biosciences, USA) and FlowJo 10 software. 293T cells transfected with empty plasmid were used as negative control. The binding activity of variants S proteins to soluble ACE2 was detected by mean fluorescence intensity (MFI) and normalized by cell surface expression of S proteins. All MFI values were weighted by multiplying by the number of positive cells in the selected gates.

**Effects of protease and endocytosis inhibitors on pseudovirus entry**

For experiments involving protease inhibitors (E64d, MCE; Camostat, MCE) or endocytosis inhibitors (Chloroquine, MCE; Apilimod, MCE), 293T-ACE2, 293T-ACE2-TMPRSS2 or Caco2-ACE2-TMPRSS2 cells were pretreated with inhibitors for 2 h before pseudovirus infection. Then, SARS-CoV-2 variants pseudoviruses were added to the respective cell culture wells. The infection efficiency of viruses was quantified by measuring luciferase activity in cell lysates.

**Cell-cell fusion assay**

To monitor the fusion activity of variants S proteins in real time, we constructed the split-GFP/Rluc8 reporter system. In brief, the spRluc8155-spGFP157 (spRG) and spGFP158-spRluc8156 (spGR) sequences were inserted into the pCAGGS vector. Then, 293T-ACE2 cells (acceptor) were transfected with pCAGGS-spGR expression plasmid and 293T cells (donor) were transfected with variants S and pCAGGS-spRG expression plasmids. 24 h post transfection, acceptor cells were trypsinized, resuspended at 2 × 10^6^/mL, treated with 60 μM EnduRen™ Live Cell Substrate, and seeded into 96-well white opaque plates at a density of 10^4^ cells/well. After 2 h of incubation, donor cells were mixed with acceptor cells in a 1:1 ratio. The mixture was then incubated in an incubator at 37°C and luciferase activity was detected at the indicated time points.

**NF- κB reporter assay**

Prior to NF-κB reporter assay, we detected the expression of variants S proteins in 293T cells by Western Blot to ensure their expression levels were similar. 293T cells (donor) were transfected with variants S proteins expression plasmids, and Caco2 cells (acceptor) were transfected with NF-κB-luc and pRL-TK (as controls) expression plasmids. After 24 h of transfection, donor and acceptor cells were mixed in a 1:1 ratio. After 12-16 h of incubation, cells were lysed and detected with dual reporter luciferase.

**Pseudovirus neutralization assay**

The effect of neutralizing mAbs and vaccine sera on the inhibition of variants pseudovirus entry was examined by measuring luciferase activity ^3,4^. In order to determine the neutralization activity of mAbs, serial 5-fold dilution of samples were prepared with the highest concentration of 10 μg/mL except for Bebtelovimab (0.5μg/mL). Similarly, serial 3-fold dilutions of vaccine sera were prepared with an initial dilution of 1:20. Diluted samples were incubated with 1000 TCID50 pseudoviruses for 1 h at 37°C, then the mixture was added to 293T-ACE2 cells. After 24 h of incubation, the neutralizing ability of mAbs and vaccine sera was tested by measuring luciferase activity in cell lysates.

**Statistical Analysis**

All data were analyzed using the GraphPad Prism 8 software. Data were presented as mean ± SEM. Half-maximal inhibitory concentration (IC50) or half-maximal inhibitory dilution (ID50) was calculated by the equation of four-parameter dose inhibition response in GraphPad Prism 8. The neutralizing activities of vaccine sera against variant pseudoviruses were evaluated using the Wilcoxon matched-pairs signed rank test. A p value less than 0.05 was defined as statistically significant (ns represents no significant difference, p < 0.05 [*], p < 0.01 [**], p < 0.001 [***], p < 0.0001 [****]).

**References**

1 Whitt, M. A. Generation of VSV pseudotypes using recombinant ΔG-VSV for studies on virus entry, identification of entry inhibitors, and immune responses to vaccines. *Journal of virological methods* **169**, 365-374 (2010).

2 Wang, R. *et al.* Analysis of SARS-CoV-2 variant mutations reveals neutralization escape mechanisms and the ability to use ACE2 receptors from additional species. *Immunity* **54**, 1611-1621 (2021).

3 Garcia-Beltran, W. F. *et al.* Multiple SARS-CoV-2 variants escape neutralization by vaccine-induced humoral immunity. *Cell* **184**, 2372-2383 (2021).

4 Li, Q. *et al.* The Impact of Mutations in SARS-CoV-2 Spike on Viral Infectivity and Antigenicity. *Cell* **182**, 1284-1294 (2020).

**Supplementary Figures**


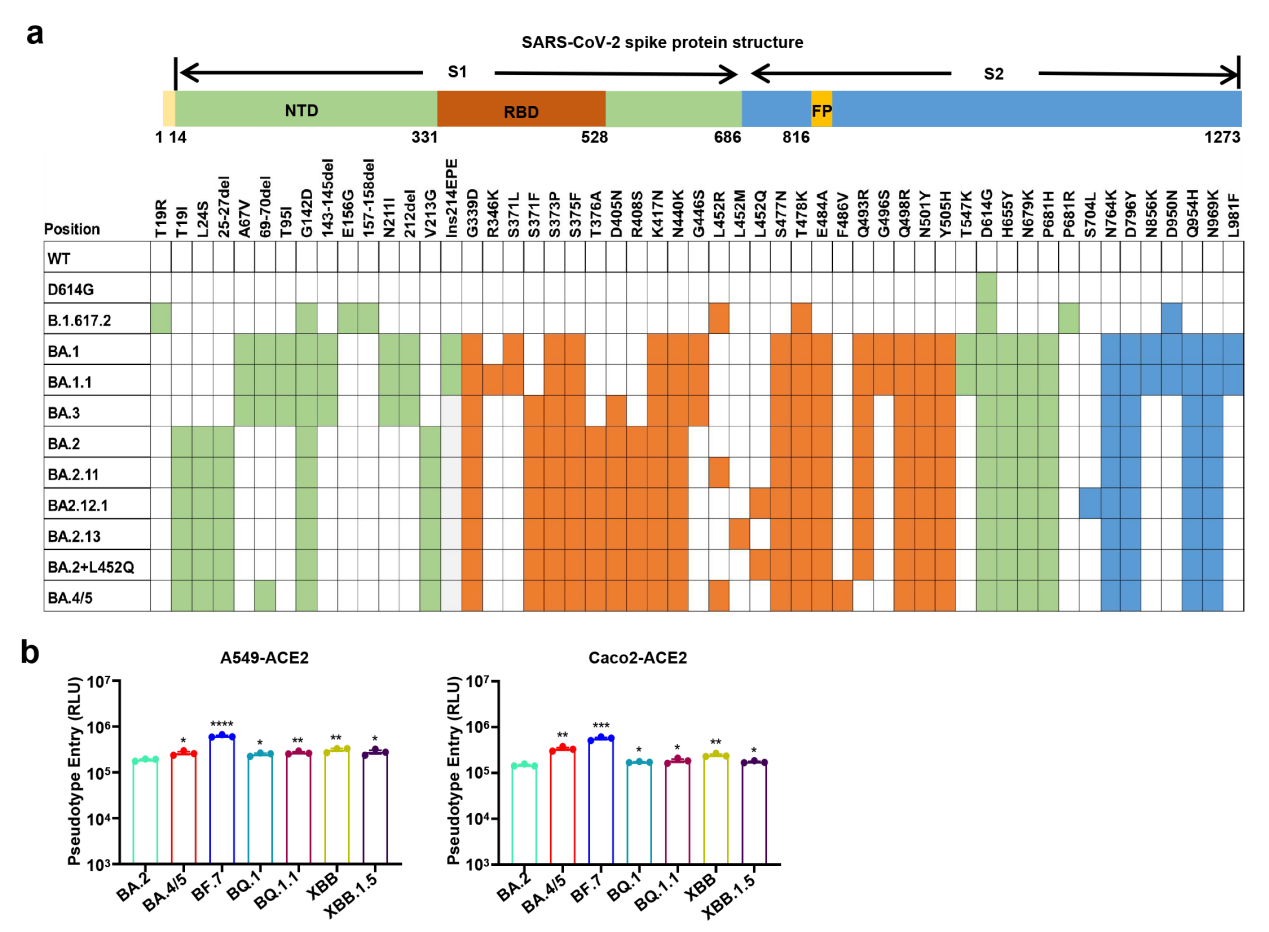


**Supplementary Fig. 1**

**a** Schematic diagram of SARS-CoV-2 spike protein structure and variants spike mutation sites.

**b** Infectivity of BA.4/5, B.7, BQ.1, BQ.1.1, XBB and XBB.1.5 pseudoviruses in A549-ACE2 and Caco2-ACE2 cells, related to Fig. 1b.

**
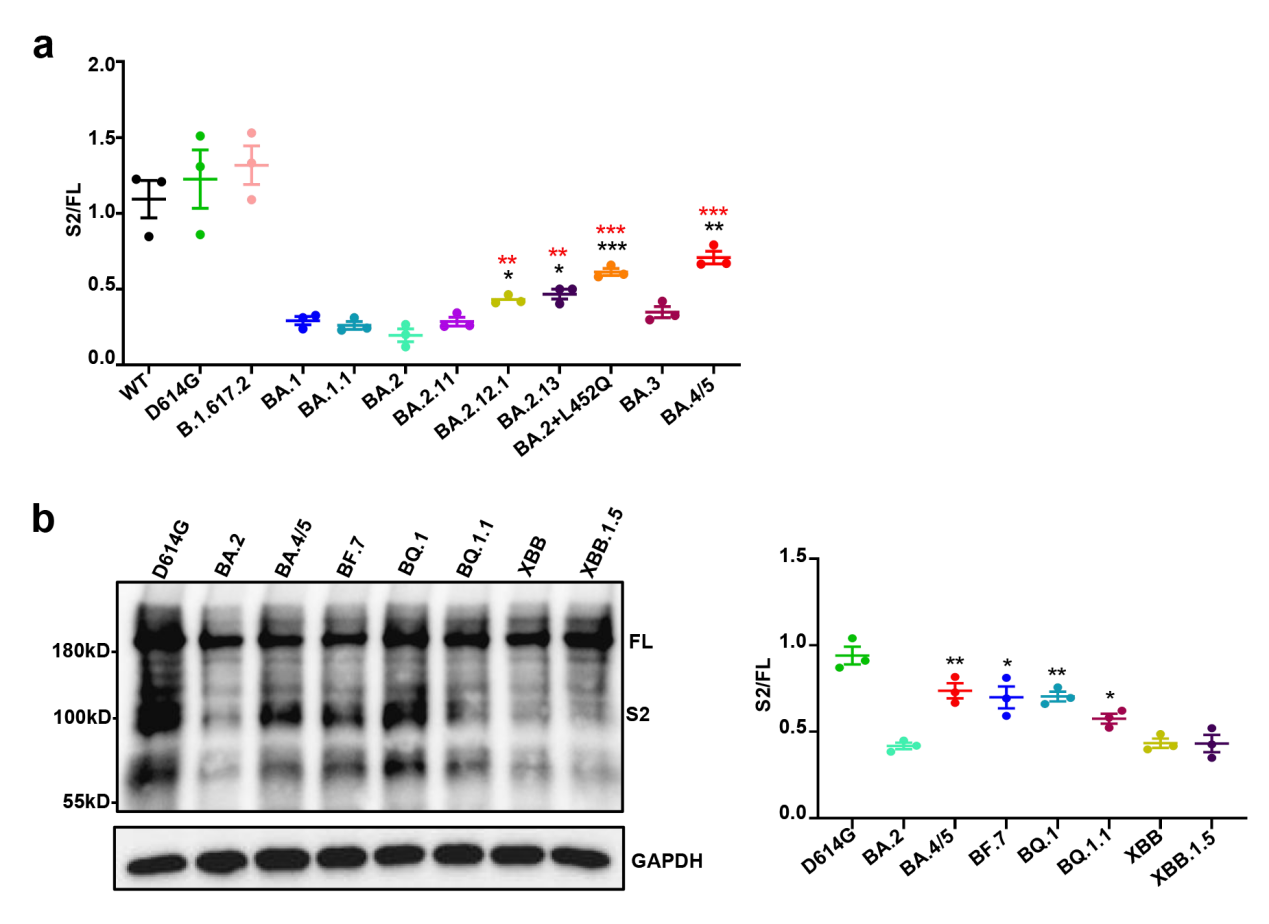
**

**Supplementary Fig. 2**

**a** Hydrolytic cleavage of variants S proteins in 293T cells, quantified by the ratio of S2 to full-length S protein, related to Figure 1c.

**b** Hydrolytic cleavage of BA.4/5, B.7, BQ.1, BQ.1.1, XBB and XBB.1.5 S proteins in 293T cells. Data are mean ± SEM of 3 replicates.

**
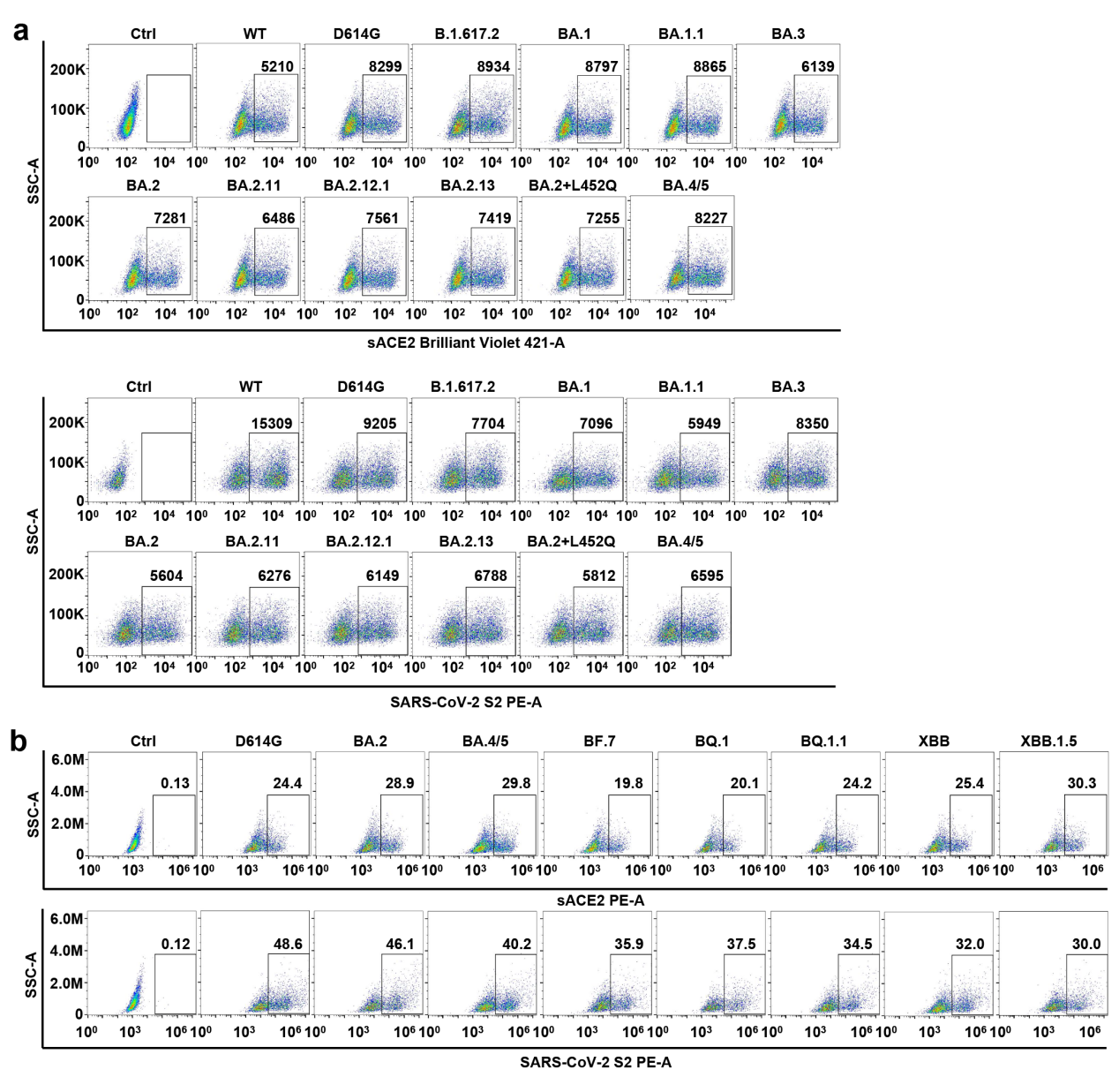
**

**Supplementary Fig. 3**

Binding to cell surface expressed variants S proteins by soluble ACE2 (S2 served as control), related to Fig. 1d, e.

**
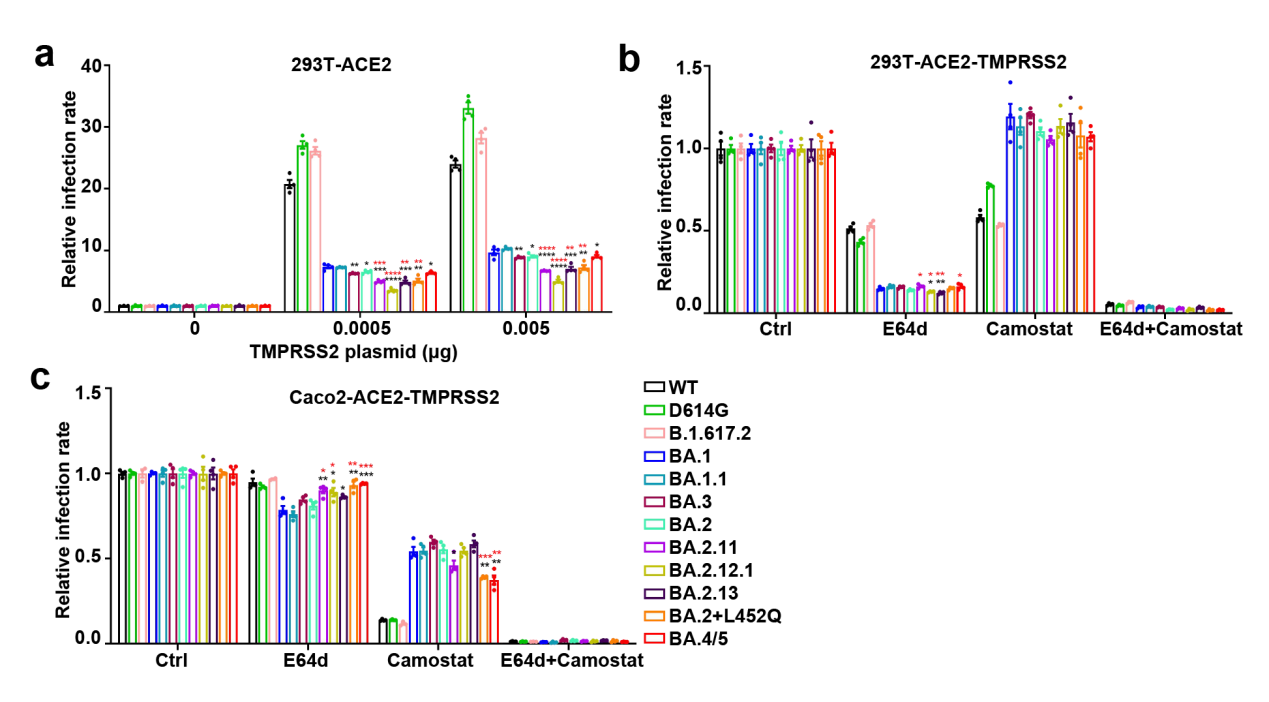
**

**Supplementary Fig. 4**

**a** Infectivity of variants pseudoviruses in 293T-ACE2 cells transiently expressed with TMPRSS2.

**b, c** Infectivity of variants pseudoviruses in 293T-ACE2-TMPRSS2 (**b**) or Caco2-ACE2-TMPRSS2 (**c**) cells pretreated with E64d (5 μM) and/or Camostat (50 μM). Black stars represent statistical differences of all Omicron sublineages compared to the BA.1 strain, while red stars represent statistical differences of all BA.2 sublineages compared to the BA.2 strain.

**Supplementary Fig. 5**

**a-c** SARS-CoV-2 variants S proteins mediated cell-cell fusion (related to Fig. 1h).

**d** Representative images of cell-cell fusion mediated by ACE2 and variants S proteins (related to Fig. 1h). Scale bar represents 200 μm. EV indicates empty vector.

**
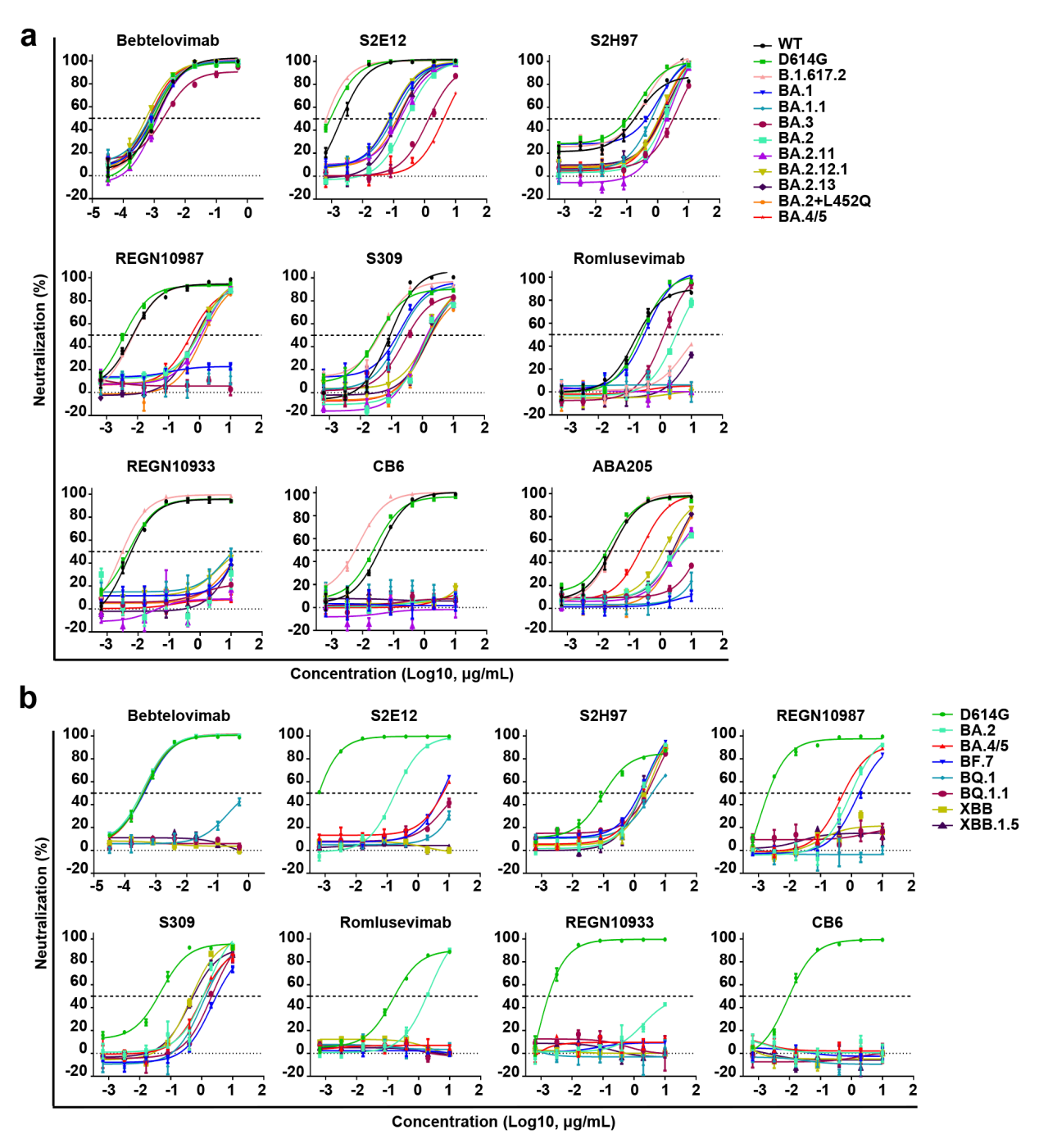
**

**Supplementary Fig. 6**

Neutralizing activity of mAbs against SARS-CoV-2 variants, related to Fig. 1j, k. Pseudoviruses carrying variants S proteins were tested against serial dilutions of each monoclonal antibody. Neutralizing activity was defined as the percent reduction in luciferase activity compared to virus control group.

**
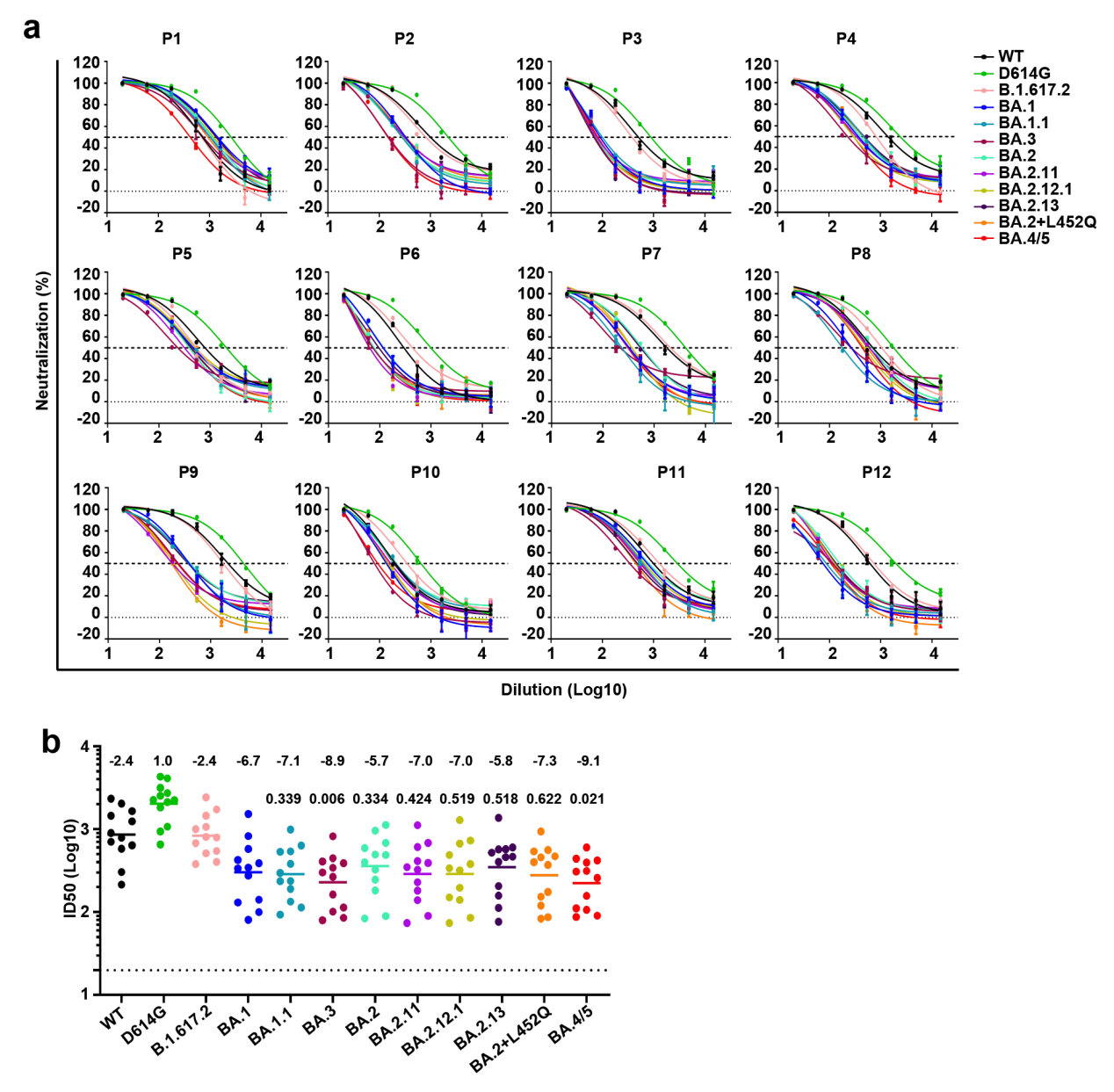
**

**Supplementary Fig. 7**

Neutralizing activity of vaccine sera against SARS-CoV-2 variants, related to Fig. 1l.

**a** Pseudoviruses carrying the indicated mutations were tested against serial dilutions of vaccine sera. Neutralization activity was defined as the percent reduction in luciferase activity relative to the virus control group.

**b** Fold changes of vaccine sera neutralization activity between variants and the D614G strain.
